# Fault Diagnosis of Wind Turbine Gearbox Based on the Optimized LSTM Neural Network with Cosine Loss

**DOI:** 10.3390/s20082339

**Published:** 2020-04-20

**Authors:** Aijun Yin, Yinghua Yan, Zhiyu Zhang, Chuan Li, René-Vinicio Sánchez

**Affiliations:** 1 State Key Laboratory of Mechanical Transmissions, Chongqing University, Chongqing 400044, China; 201807021027@cqu.edu.cn (Y.Y.); 20170702004t@cqu.edu.cn (Z.Z.); 2 College of Mechanical Engineering, Chongqing University, Chongqing 400044, China; 3 Research Center of System Health Maintenance, Chongqing Technology and Business University, Chongqing 400067, China; chuanli@ctbu.edu.cn; 4 Department of Mechanical Engineering, Universidad Politécnica Salesiana, Cuenca 010105, Ecuador; rsanchezl@ups.edu.ec

**Keywords:** wind turbine, gearbox fault, cosine loss, long short-term memory network

## Abstract

The gearbox is one of the most fragile parts of a wind turbine (WT). Fault diagnosis of the WT gearbox is of great importance to reduce operation and maintenance (O&M) costs and improve cost-effectiveness. At present, intelligent fault diagnosis methods based on long short-term memory (LSTM) networks have been widely adopted. As the traditional softmax loss of an LSTM network usually lacks the power of discrimination, this paper proposes a fault diagnosis method for wind turbine gearboxes based on optimized LSTM neural networks with cosine loss (Cos-LSTM). The loss can be converted from Euclid space to angular space by cosine loss, thus eliminating the effect of signal strength and improve the diagnosis accuracy. The energy sequence features and the wavelet energy entropy of the vibration signals are used to evaluate the Cos-LSTM networks. The effectiveness of the proposed method is verified with the fault vibration data collected on a gearbox fault diagnosis experimental platform. In addition, the Cos-LSTM method is also compared with other classic fault diagnosis techniques. The results demonstrate that the Cos-LSTM has better performance for gearbox fault diagnosis.

## 1. Introduction

With the gradual depletion of non-renewable energy and the deteriorating human living environment, wind energy has developed rapidly as one renewable energy source [1]. However, wind turbines (WTs) are mostly installed in remote areas as the main equipment for wind power generation. The harsh operating environment causes frequent failures of key components such as gearboxes and bearings [2]. Therefore, in order to ensure the safe operation of WTs and reduce the operation and maintenance (O&M) costs, it is crucial to study effective fault diagnosis methods for gearboxes [3].

As the vibration and acoustic emission signals are sensitive to the faults of the machine, condition monitoring systems based on vibration [4,5] and acoustic emission [6,7,8] have been widely used in the field of condition monitoring and fault diagnosis. In order to monitor the health conditions of WTs, the wind energy industry is currently using condition monitoring systems to collect large amounts of real-time data for diagnosing gearbox faults. Since the amount of data collected from gearboxes is increasing, the traditional fault diagnosis method cannot effectively analyze massive data and automatically give accurate diagnosis results [9]. Therefore, intelligent fault diagnosis methods based on artificial intelligence techniques are gaining more attention. Generally, there are two main steps for intelligent fault diagnosis methods: feature extraction and fault classification [10]. Traditional methods such as artificial neural networks (ANN) and support vector machine (SVM) are used to classify faults [11,12,13]. However, the problem of existing intelligent fault diagnosis methods is that the common machine learning methods rely on well-selected features and have limited ability to learn from complex time-series signals; meanwhile, with these methods it is more difficult to identify faults under variable working conditions, and they have a low classification accuracy. Therefore, a more effective fault identification method is needed. [14,15,16,17]. In recent years, deep learning has attracted great attention from various fields due to the powerful ability of feature learning and the superiority of processing massive data. Up to now, deep learning networks have been widely applied in fault diagnosis, such as deep belief networks (DBN) [18], convolutional neural networks (CNN) [19] and recurrent neural networks (RNN) [20]. However, the gearbox has strong time-dependence of faults due to its relatively long operating time [21]. Compared with other deep learning methods, the long short-term memory (LSTM) neural network has great advantages in learning long-term time-dependent characteristics of sequences [22,23].

For the fault diagnosis methods based on LSTM neural networks, the softmax cross entropy is usually used as the loss function of fault classification. However, recent studies found that the traditional softmax loss is insufficient to acquire the discriminating power for classification. To obtain better discriminating performance, Wang et al. [18] proposed a novel loss function called large margin cosine loss (LMCL) for learning the high-resolution depth features used in face recognition. The result shows that the loss function based on cosine distance has a good effect on classification. Therefore, this paper proposes an optimized fault diagnosis method using an LSTM network with cosine loss (Cos-LSTM) to improve the ability of classification. Meanwhile, the energy sequence features and the wavelet energy entropy of the fault vibration data collected on a gearbox fault diagnosis experimental platform are used to validate Cos-LSTM networks. The Cos-LSTM achieves higher accuracy of diagnosis, which is demonstrated through the gear transmission experiments and compared to other fault diagnosis methods.

The rest of the paper is organized as follows. In Section 2, the typical architecture of LSTM and the process of fault diagnosis are briefly introduced. Section 3 details the Cos-LSTM method and the process of gearbox fault diagnosis based on the Cos-LSTM method. The gearbox fault diagnosis experiment and the comparisons of our proposed method and other fault diagnosis methods are presented in Section 4. Finally, the conclusions are drawn in Section 5.

## 2. LSTM Neural Network for Fault Diagnosis

As a special type of recurrent neural network (RNN), the LSTM neural network was proposed by Hochreiter and Schmifhuber [24] to solve the vanishing or exploding gradient problem of RNNs [25], while retaining the ability of RNNs to process sequential data. In this section, we describe LSTM in more detail.

### 2.1. Structure of LSTM 

**Figure 1:**
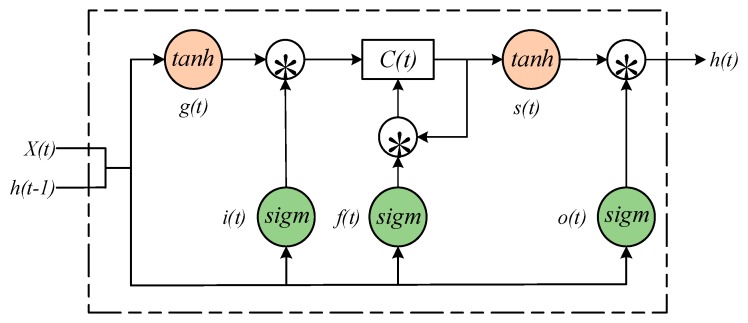
The Schematic diagram of an LSTM cell.

The main component of an LSTM neural network is the LSTM cell, which can decide whether to update the state information of a memory cell. The structure of the LSTM cell is shown in Figure 1.

As shown in Figure 1, *h*(*t*) and *x*(*t*) are the output hidden states and inputs of the current time step, *h*(*t* − 1) represents the hidden state of the previous time step; sigm is the sigmoid function and *tanh* is the hyperbolic tangent function. *C*(*t*) is a memory cell which is used for the preservation of information, and the flow of information into or out of *C*(*t*) is regulated by three different gates:

1. The input gate *i*(*t*)*,* which decides whether the information can get in the memory element;
2. The forget gate *f*(*t*), which decides whether the internal information needs to be forgotten;
3. The output gate *o*(*t*), which decides what information can pass through the gate and get into the rest of the neural network.

The internal state node *s*(*t*) and input node *g(t*) are also integral parts of the LSTM cell. Here are the calculation procedures of the LSTM cell:

(1) $$g(t)=\Phi (W_{gx}x(t)+W_{gh}h(t-1)+b_{g}),$$

(2) $$i(t)=\sigma (W_{ix}x(t)+W_{ih}h(t-1)+b_{i}),$$

(3) $$f(t)=\sigma (W_{fx}x(t)+W_{fh}h(t-1)+b_{f}),$$

(4) $$o(t)=\sigma (W_{ox}x(t)+W_{oh}h(t-1)+b_{o}),$$

(5) s(t)=g(t)∗i(t)+C(t−1)∗f(t),

(6) h(t)=Φ(s(t))∗o(t).

In the above equations, *W_jx_*, *W_jh_* and *b_j_*, j=g,j,f,o denote the input weight matrixes, hidden weight matrixes and bias vectors separately; *∗, σ* and Φ are element-wise multiplications of two vectors, the *sigmoid* function and *tanh* function, respectively. 

The LSTM neural network can learn when to open or close the gate to control the flow of information in LSTM cells automatically, so it can choose useful information to train the model.

### 2.2. Architecture of LSTM for Fault Diagnosis

**Figure 2:**
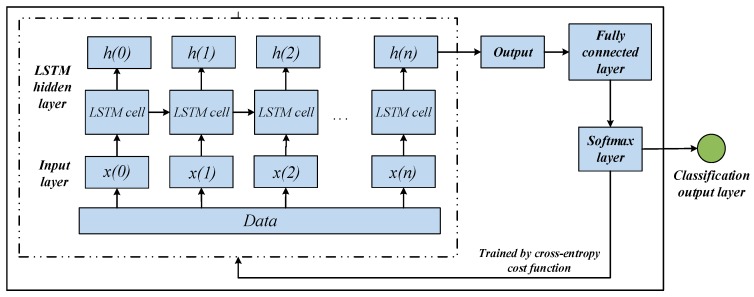
The architecture for LSTM network.

The LSTM neural network is used for fault classification in fault diagnosis. The architecture for the LSTM network includes five layers: an input layer, an LSTM hidden layer, a fully connected layer, a softmax layer and a result output layer at the end. The architecture of the LSTM network is shown in Figure 2.

During the training process, the fault features are fed into the input layer first, then the data flow through LSTM cell and the result of LSTM cell is output to the LSTM hidden layer. The last output of the LSTM hidden layer is taken as the output of the LSTM network, and it is used to connect a fully connected layer to map outputs into the result space. The softmax layer follows the fully connected layer to calculate the probabilities for all the fault pattern. Finally, the fault diagnosis results are output to the classification output layer. After completing the training, the weights and bias will be adjusted to the optimal value, and then the test set is input into LSTM for fault diagnosis. 

## 3. Cos-LSTM

The softmax cross entropy is often used as the loss function of the LSTM neural network; however, the softmax loss is insufficient to enable classification [26,27]. To solve this problem, the cosine loss function is adopted to optimize the LSTM neural network. This section provides details about the Cos-LSTM.

### 3.1. Cosine Loss

**Figure 3:**
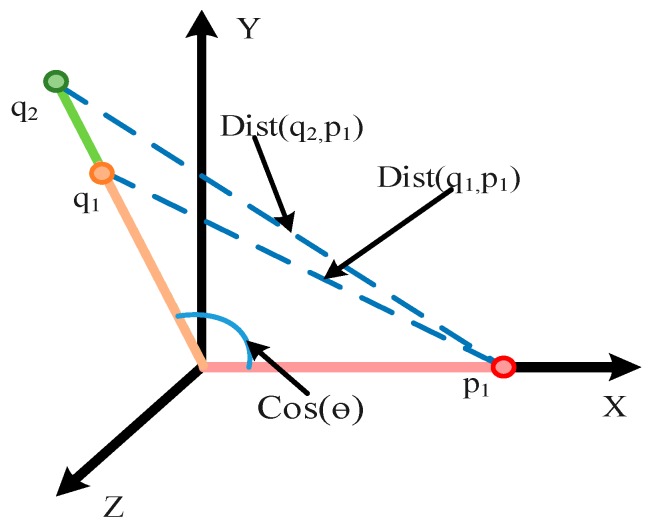
Schematic of Cosine Loss.

Based on the softmax loss, the cosine loss retains its advantage of enlarging the difference between classes [15], but reduces its sensitivity to different signal strengths and pays more attention to the difference of vectors in direction. The schematic of cosine loss is shown in Figure 3. 

Suppose there are two signals $q_{1}$ and $q_{2}$ with the same fault, and the corresponding fault label is $p_{1}$. When softmax is taken as the loss function, the softmax loss can be formulated as follows,

(7) $$L{\mathrm{oss}}_{\mathrm{soft}}=\frac{1}{B}\sum_{i=1}^{B}-\log(\frac{e^{∥W_{i}∥∥x∥\cos\theta_{i}}}{\sum_{j=1}^{N}e^{∥W_{j}∥∥x∥\cos\theta_{j}}})$$

where *B* is the number of training samples and *N* is the number of classes, *x* and W represent the hidden layer output and the weight matrix respectively, and *θ* is the angle between W and *x.* Formula (2) suggests that softmax loss is related to signal strength, while cosine loss evaluates the size of the differences between classes according to cosine similarity between the two feature vectors. The cosine similarity is defined as follows:

(8) $$similarity(A,B)=\frac{A\cdot B}{∥A∥*∥B∥}=\frac{\sum_{i=1}^{n}A_{i}*B_{i}}{\sqrt{\sum_{i=1}^{n}A_{i}^{2}}*\sqrt{\sum_{i=1}^{n}B_{i}^{2}}}$$

Taking 1—cosine similarity as the loss function, the cosine loss can be formulated as follows,

(9) $$L{\mathrm{oss}}_{\cos}=\frac{1}{B}\sum_{i=1}^{B}1-\frac{y_{i}}{\sqrt{\sum_{j=1}^{N}y_{j}^{2}}}=\frac{1}{B}\sum_{i=1}^{B}\sqrt{1-\frac{∥W_{i}{∥}^{2}∥x{∥}^{2}\cos{\theta_{i}}^{2}}{\sum ∥W_{j}{∥}^{2}∥x{∥}^{2}\cos{\theta_{j}}^{2}}}$$

By Formula (5), the $∥x{∥}^{2}$ in this formula can be eliminated, so the cosine loss is independent of the signal strength. Therefore, taking cosine loss function as the loss function in gearbox fault diagnosis, the loss can be converted from Euclid space to angular space, thus eliminating the effect of signal strength and reduce the burden of network fitting.

### 3.2. The Process of Cos-LSTM for Fault Diagnosis

In this paper, there are two kinds of fault features extracted for evaluating the proposed method: the energy sequence feature and the wavelet energy entropy.

The energy sequence feature: The energy sequence features are extracted by wavelet packet decomposition (WPD). WPD is a signal decomposition tool that decomposes a signal to some nodes and every node represents a set of coefficients at a specified frequency band [28,29]. The wavelet packet is defined as follows:

(10) $$\phi (t)=\sqrt{2}\sum_{k}h(k)\phi (2t-1)$$

(11) $$\Psi (t)=\sqrt{2}\sum_{k}g(k)\phi (2t-1)$$

where h(k) and g(k) are a low-pass filter and a high-pass filter respectively. ∅(t) and Ψ(t) represent the scaling function and the wavelet function respectively. Additionally, g(k) can be expressed by h(k) using the formula $g\left(k\right)={\left(-1\right)}^{k}h\left(1-k\right)$. 

The signal is decomposed by Equations (12) and (13)

(12) $$d_{j+1,2n}(t)=\sum_{l\in Z}h_{l-2k}d_{j,n}(t)$$

(13) $$d_{j+1,2n+1}(t)=\sum_{l\in Z}g_{l-2k}d_{j,n}(t)$$

where *j* denotes the decomposition layer, $n\in \left\{0,1,2,\ldots ,2^{j}-1\right\}$ is the number of nodes in layer *j*, *l* indicates the number of wavelet coefficients and $d_{j,n}$ represents the coefficient sequence at the *j*th layer, *n*th node.

Due to the large amount of data, we divided the vibration data into four segments and a three-layer WPD was performed on each segment of vibration data using Daubechies 3 (db 3) to obtain eight nodes [30,31,32]. The energy of each node $E_{j,n}$ could then be calculated through Formula (14)

(14) $$E_{j,n}=\sum_{k}|d_{j,n}(k){|}^{2}.$$

The total energy of the signal *E* is the sum of the energy of each node in layer three. It can be computed by (15):

(15) $$E=\sum_{n=0}^{2^{3}-1}E_{j,n}.$$

and $P_{j,n}$ is defined by (16):

(16) $$P_{j,n}=\frac{E_{j,n}}{E}.$$

Each of the signals can be decomposed to get eight nodes, and the energy sequences feature can be expressed as Equation (17) according to Equations (14)–(16).

(17) $$x(i)=(P_{2,i}^{v1},P_{2,i}^{v2})$$

where x(i) is the energy sequences feature and i=0,1,…,7, $P_{2,i}^{v1}\;\mathrm{and}\;P_{2,i}^{v2}$ indicate the *P*_2,*i*_ for $s_{v1}\left(t\right)\;\mathrm{and}\;s_{v2}\left(t\right)$, which denote the vibration signals of the gearbox in the horizontal and vertical directions respectively. 

Wavelet energy entropy: The signal is reconstructed according to the eight node coefficients obtained from the three-layer WPD above, and the reconstructed signal is divided into *N* segments on the basis of the time characteristics of the signal. The energy of each segment is calculated by Formula (14). The calculated energy is normalized by Formulas (15) and (16) to obtain the wavelet energy entropy. The wavelet energy entropy of the *j*-th layer *n* node of the WPD is defined as $H_{j,n}$, and can be formulated as follows:

(18) $$H_{j,n}=-\sum_{i=1}^{N}P_{j,n}(i)\log P_{j,n}(i)$$

where $P_{j,n}\left(i\right)$ is the normalized value of the energy of each segment of the signal; i=0,1,…,N. The value of *N* is 50 in this article.

According to the calculated wavelet energy entropy of each node, the wavelet energy entropy feature is formed by Equation (19):

(19) $$T=\left[H_{3,1},H_{3,2},H_{3,3},H_{3,4},H_{3,5},H_{3,6},H_{3,7},H_{3,8}\right]$$

**Figure 4:**
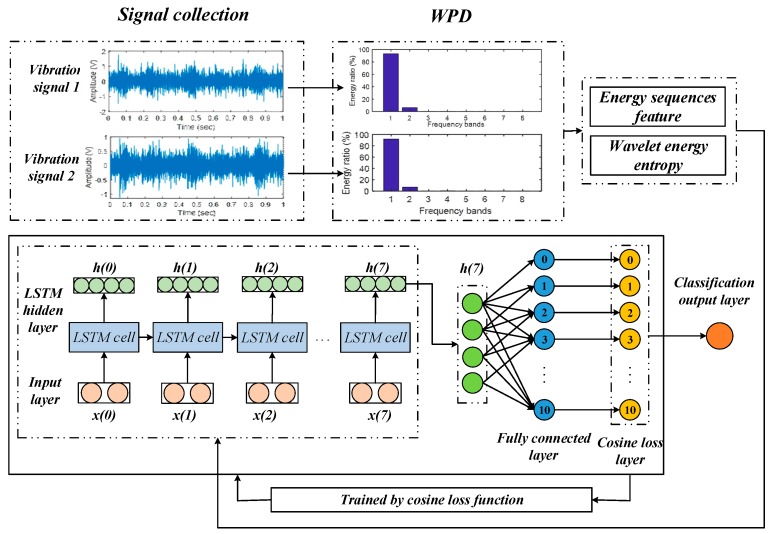
The flow chart of the Cos-LSTM method for gearbox fault diagnosis.

The fault features obtained above are fed into the Cos-LSTM network to diagnose the gearbox fault. The flow chart of fault diagnosis based on the Cos-LSTM is shown in Figure 4. 

We used one LSTM hidden layer with eight LSTM cells to extract deeper features. The fault features are first normalized and then fed into the input layer. In this paper, we used *N* samples (*N* = 2200 samples) to train the model. Therefore, the size of the input layer is *N* × 8 (time steps) × 2 (2-dimensional features), and the input size of each LSTM cell is *N* × 2. The last output *h* (7) of the LSTM hidden layer connects a fully connected layer with 11 neurons, using cosine loss to calculate the probabilities for the 11-fault pattern.

**Figure 5:**
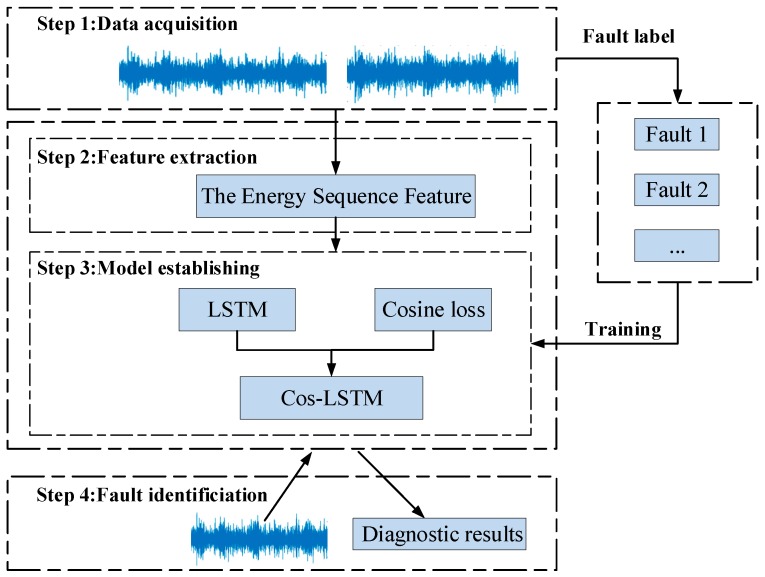
The workflow of the Cos-LSTM.

The parameters of the LSTM neural network are presented as follows: time steps for LSTM = 8; the LSTM hidden layer neurons = 4; the fully connected layer neurons = 11; learning rate = 0.01; number of iterations of training = 10,000. The workflow of the Cos-LSTM is shown in Figure 5.

## 4. Experimental Validation

### 4.1. Experiment Description

**Figure 6:**
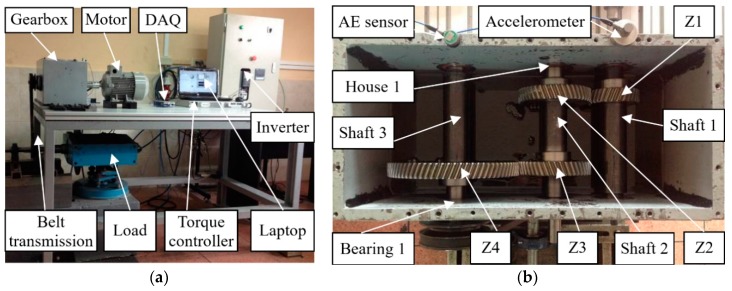
(**a**) Experimental test rig and (**b**) the structure of the gearbox.

The experimental test rig is illustrated in Figure 6a,b. The motor was controlled by an inverter and connected to the input shaft of the gearbox to transmit power by a coupling. An electromagnetic torque load was coupled with the output shaft of the gearbox through a V-belt. The electromagnetic torque load was controlled by a torque controller (TDK-Lambda, GEN 100-15-IS510; TDK-Lambda, Wuxi, China), which can adjust the torque of the load manually. Two accelerometers were mounted on the gearbox to collect signals, and the signals collected were transmitted to a laptop using the data acquisition card. Detailed information on the data acquisition system is provided in Table 1.

The structure of the gearbox is displayed in Figure 6b. It consists of four gears, six bearings and three shafts. Shaft 1 was the input shaft, and gear Z1, with a module of 2.25 mm, a pressure angle of 20, a helical angle of 20, and 30 teeth, was installed on it. Shaft 1 transmitted the power to shaft 2 by a pair of gears (Z1 and Z2) in mesh. The output shaft (shaft 3) was driven by another helical gear Z4, with 80 teeth, which was meshed with the gear Z3. The helical gears Z2 and Z3 installed on shaft 2 both have 45 teeth and other parameters of them are the same to Z1. We installed one of the faulty components: bearing 1, bearing house 1, and gears Z1, Z2, Z3, Z4 every time on the gearbox to experiment. Table 2 shows all the condition patterns of the gearbox.

### 4.2. Experimental Results

**Figure 7:**
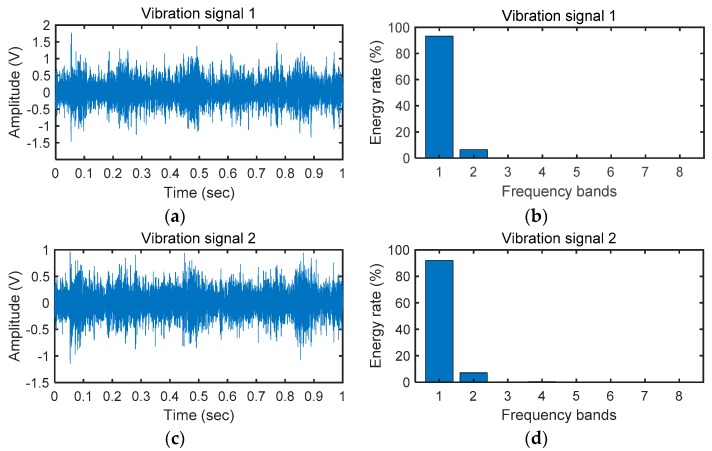
The raw vibration signals and energy distribution map.

Firstly, we verified the Cos-LSTM with the energy sequence features. We chose a test sample for explanation of the fault diagnosis process of our proposed method. The pattern number of this sample is 3 (chafing tooth), and the input speed and load of this sample are set to 480 rpm and zero respectively. The raw vibration signals and energy distribution map is shown in Figure 7. Figure 7a,c presents the raw signals $s_{v1}\;\left(t\right),\;s_{v2}\;\left(t\right)$ of this sample collected on the gearbox and Figure 7b,d presents their energy distribution maps of the third layer WPD $P^{v1}$ and $P^{v2}$. Putting the energy sequences feature of this sample into the Cos-LSTM, we got the probability of each fault pattern for the sample. The probability of the no. 3 fault pattern is 99.97% and the other 10 faults have a probability of 0.03%. The result shows that our proposed method considers that there is a fault numbered 3 (chafing tooth) in the gearbox. The result is correct for this test sample, so the method we proposed is effective.

**Figure 8:**
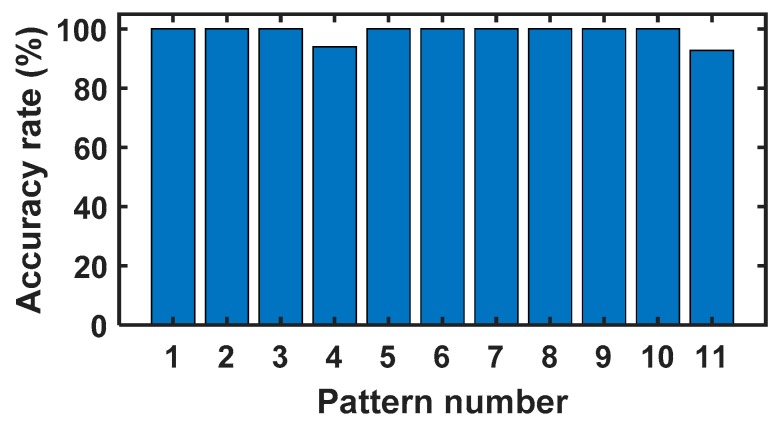
The accuracy rates for the 11 fault patterns.

**Figure 9:**
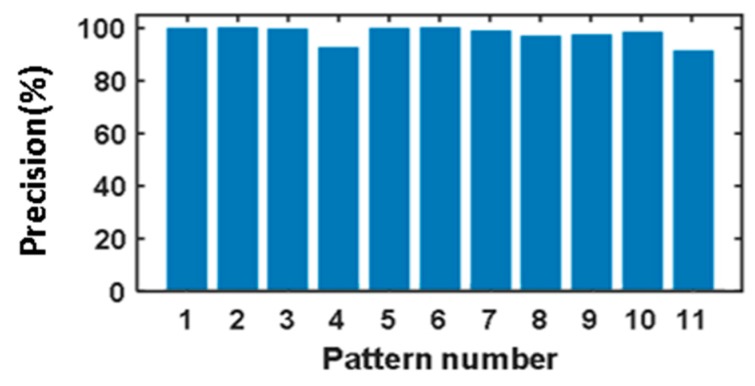
The precision for the 11 fault patterns.

From Table 2, it can be seen that three different input speeds and loads are set for all 11 fault patterns. Therefore, we have a total of 99 different tests, and each test is repeated five times. In each test, the signals are collected with 10 durations, and every duration covers 1 s. Therefore, we can get 9900 vibration signals. In order to train the model, we randomly choose 2200 samples as the training dataset. With the trained model, another 550 randomly chosen samples are used to test the effectiveness of the model. The effectiveness is measured by the accuracy rate. In this experiment, the accuracy rate is the number of correctly diagnosed samples divided by all the test samples, and the precision is the ratio of the number of samples correctly diagnosed with a fault pattern to the total number of samples diagnosed with such a fault pattern. The accuracy rate of the model is 98.55% in 550 samples. The accuracy rates and precision of our proposed model for the 11 fault patterns are shown in Figure 8 and Figure 9 respectively.

### 4.3. Comparison Analysis

In this paper, the energy sequence features were used to verify the superiority of the Cos-LSTM by comparing with the traditional LSTM based on softmax loss and classic fault diagnosis methods, such as SVM, K-nearest neighbor (KNN) and backpropagation (BP) neural networks. In order to better evaluate the accuracy of the Cos-LSTM, we also used wavelet energy entropy feature for the fault diagnosis test. Table 3 shows the comparison results. Meanwhile, the different energy sequence features were extracted by changing the parameters of WPD such as wavelet basis function and data segment size, for evaluating the accuracy of the Cos-LSTM, and the results are displayed in Table 4.

According to Table 3 and Table 4, the Cos-LSTM has the highest accuracy rate (98.55%) compared to other methods in the experimental results on the energy sequence features. After comparison and analysis, it can be found that: (1) comparison with traditional LSTM shows that the classification ability of cosine loss is better than that of softmax loss; (2) the accuracy rate of the LSTM neural network is better than KNN, SVM and BP neural networks, which indicates that the LSTM neural network has better feature-learning ability compared to classic fault diagnosis methods; (3) the evaluation results of Cos-LSTM using wavelet energy entropy are close to those using energy sequence features; (4) the accuracy rate of the Cos-LSTM is influenced by the energy sequence features extracted with different parameters of WPD, and the result shows that the energy sequence features extracted based on the wavelet basis function of Daubechies 3 (db3) and segment size 4 have better diagnostic accuracy rates; and (5) combined with the experimental results of energy sequence features and wavelet energy entropy, Cos-LSTM is able to diagnose the faults of the gearbox effectively. 

## 5. Conclusions

This paper presented a fault diagnosis method for WT gearboxes based on the optimized LSTM network with cosine loss. The energy sequence features and the wavelet energy entropy were used to evaluate the Cos-LSTM network. The effectiveness of the Cos-LSTM was verified by a fault diagnosis experiment on a gearbox. The classification results show that the performance of the Cos-LSTM is better than that of the traditional LSTM and classic fault diagnosis techniques. Thus, the proposed method has superior performance in fault diagnosis. In the future, new studies will be conducted on feature learning directly from raw vibration signals using LSTM neural networks. 

## Figures and Tables

**Table 1:** Data acquisition settings.

Item	Parameter
Sensor	PCB ICP 353C03 accelerometer
Data acquisition box Software	NI cDAQ-9234 LabVIEW
Sampling rate	50 kHz

**Table 2:** Condition patterns of the gearbox.

Pattern Number	Faulty Component	Faulty Name	Input Speed (rpm)	Load (V)	View of the Failure
1	N/A	N/A	480, 720, 900	0, 10, 30	N/A
2	Gear *Z*_1_	Worn tooth	480, 720, 900	0, 10, 30	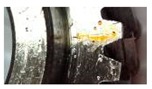
3	Gear *Z*_2_	Chafing tooth	480, 720, 900	0, 10, 30	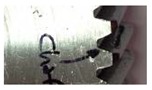
4	Gear *Z*_3_	Pitting tooth	480, 720, 900	0, 10, 30	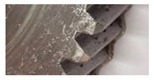
5	Gear *Z*_3_	Worn tooth	480, 720, 900	0, 10 30	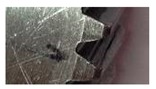
6	Gear *Z*_4_	Root crack tooth	480, 720, 900	0, 10, 30	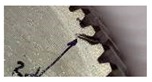
7	Gear *Z*_4_	Chafing tooth	480, 720, 900	0, 10, 30	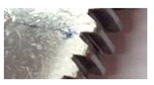
8	Bearing 1	Inner race fault	480, 720, 900	0, 10, 30	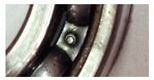
9	Bearing 1	Outer race fault	480, 720, 900	0, 10, 30	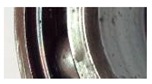
10	Bearing 1	Ball fault	480, 720, 900	0, 10, 30	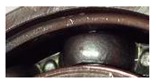
11	House 1	Eccentric	480, 720, 900	0, 10, 30	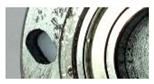

**Table 3:** Comparisons with other classic fault diagnosis methods.

Feature	Fault Diagnosis Methods	Accuracy Rate
The energy sequence	Cos-LSTM	98.55%
	LSTM	96.72%
	SVM	65.48%
	KNN	83.93%
	BP neural network	69.64%
Wavelet Energy entropy	Cos-LSTM	98.08%

**Table 4:** Comparisons with different parameter of WPD.

Item	Parameter	Accuracy Rate
Wavelet basis function	Daubechies 3	98.55%
	Daubechies 2	96.36%
	Haar	93.82%
	Symlet	97.09%
Segment size	2	96.63%
	3	97.12%
	4	98.55%
